# Sulthiame impairs mitochondrial function in vitro and may trigger onset of visual loss in Leber hereditary optic neuropathy

**DOI:** 10.1186/s13023-021-01690-y

**Published:** 2021-02-04

**Authors:** Marie-Christine Reinert, David Pacheu-Grau, Claudia B. Catarino, Thomas Klopstock, Andreas Ohlenbusch, Michael Schittkowski, Ekkehard Wilichowski, Peter Rehling, Knut Brockmann

**Affiliations:** 1 Division of Pediatric Neurology, Department of Pediatrics and Adolescent Medicine, University Medical Center Göttingen, Robert-Koch-Str. 40, 37075 Göttingen, Germany; 2 Department of Cellular Biochemistry, University Medical Center Göttingen, Göttingen, Germany; 3 Department of Neurology, Friedrich-Baur-Institute, University Hospital, LMU Munich, Munich, Germany; 4 German Center for Neurodegenerative Diseases (DZNE), Munich, Germany; 5 Munich Cluster for Systems Neurology (SyNergy), Munich, Germany; 6 Department of Ophthalmology, Section for Strabismus, Neuroophthalmology and Oculoplastics, University Medical Center Göttingen, Göttingen, Germany; 7 Cluster of Excellence “Multiscale Bioimaging: From Molecular Machines To Networks of Excitable Cells” (MBExC), University of Göttingen, Göttingen, Germany; 8 Max Planck Institute for Biophysical Chemistry, Göttingen, Germany; 9 Interdisciplinary Pediatric Center for Children With Developmental Disabilities and Severe Chronic Disorders, University Medical Center Göttingen, Göttingen, Germany

**Keywords:** Sulthiame, Carbonic anhydrase inhibitor, Adverse effects, Leber hereditary optic neuropathy, LHON, Oxygen consumption rate

## Abstract

**Background:**

Leber hereditary optic neuropathy (LHON) is the most common mitochondrial disorder and characterized by acute or subacute painless visual loss. Environmental factors reported to trigger visual loss in LHON mutation carriers include smoking, heavy intake of alcohol, raised intraocular pressure, and some drugs, including several carbonic anhydrase inhibitors. The antiepileptic drug sulthiame (STM) is effective especially in focal seizures, particularly in benign epilepsy of childhood with centrotemporal spikes, and widely used in pediatric epileptology. STM is a sulfonamide derivate and an inhibitor of mammalian carbonic anhydrase isoforms I–XIV.

**Results:**

We describe two unrelated patients, an 8-year-old girl and an 11-year-old boy, with cryptogenic focal epilepsy, who suffered binocular (subject #1) or monocular (subject #2) visual loss in close temporal connection with starting antiepileptic pharmacotherapy with STM. In both subjects, visual loss was due to LHON. We used real-time respirometry in fibroblasts derived from LHON patients carrying the same mitochondrial mutations as our two subjects to investigate the effect of STM on oxidative phosphorylation. Oxygen consumption rate in fibroblasts from a healthy control was not impaired by STM compared with a vehicle control. In contrast, fibroblasts carrying the m.14484T>C or the m.3460G>A LHON mutation displayed a drastic reduction of the respiration rate when treated with STM compared to vehicle control.

**Conclusions:**

Our observations point to a causal relationship between STM treatment and onset or worsening of visual failure in two subjects with LHON rather than pure coincidence. We conclude that antiepileptic medication with STM may pose a risk for visual loss in LHON mutation carriers and should be avoided in these patients.

## Background

The antiepileptic drug sulthiame (STM), *N*-(4-sulfamoylphenyl)-1,4-butansultam, was developed in the 1950s and introduced in the market in the 1960s. STM proved to be effective especially in focal seizures, particularly in benign epilepsy of childhood with centrotemporal spikes (BECTS) [1–4]. Moreover, several open prospective and retrospective studies showed efficacy of STM as add-on treatment in cryptogenic or symptomatic localization-related epilepsies in children and adults [2]. For treatment of BECTS and related epileptic syndromes a dose of 4 to 8 mg/kg body weight (BW) per day is recommended, and higher doses in West syndrome.

STM is available in Argentina, Australia, Czech Republic, Germany, Greece, Latvia, Hungary, Israel, Slovakia, and Switzerland [2]. It is widely used in genetic, cryptogenic, and symptomatic focal epilepsies, especially in childhood and adolescence [2].

Several studies demonstrated a linear correlation between the STM dose/BW and STM serum concentrations in children and adults [1, 4].

STM is a sulfonamide derivate [4]. Alike the antiepileptic drugs zonisamide and topiramate, STM is an inhibitor of mammalian carbonic anhydrase isoforms I-XIV [5]. Carbonic anhydrase inhibitors may cause bone marrow depression, skin toxicity, sulfonamide-like renal lesions, and allergic reactions in hypersensitive individuals. Dependent on the dosage patients may show drowsiness and paresthesias. Largely, adverse effects are secondary to urinary alkalinization or metabolic acidosis [6, 7].

Well-known adverse effects of STM comprise paraesthesias of face and limbs, exercise hyperpnoea, dyspnoea, and anorexia. Rare side effects include cognitive impairment, elevation of liver enzymes, acute renal failure, and metabolic acidosis [8–13]. Visual loss or any optic nerve damage were never reported as a side effect of STM.

Here, we report two unrelated children carrying two different mutations of mitochondrial DNA associated with Leber hereditary optic neuropathy (LHON), who suffered visual loss in close temporal connection with STM medication for focal epilepsy. We discuss whether this is pure coincidence or reflects STM being a trigger factor for onset of visual failure in LHON mutation carriers. Based on biochemical investigations in fibroblasts we provide evidence for marked impairment of oxidative phosphorylation caused by STM.

## Results

### Subjects

#### Subject 1

This currently 15-year-old girl is the only child of healthy non-consanguineous parents. Several maternal relatives had suffered visual loss in young adult age and had been diagnosed with LHON. After uneventful pregnancy she was born at term with normal weight, length, and head circumference. A small omphalocele, noted postnatally, was closed surgically. Her motor, verbal, and cognitive development was normal.

At age 4 years 6 months, her first ophthalmological examination revealed visual acuity (VA) of 0.5 to 0.6 on both eyes, which may be appropriate for this age, and normal color vision. Funduscopy showed a normal vital optic nerve head bilaterally.

At 5 years of age she presented with recurrent episodes of headache, vertigo, vomiting, and ataxia with sudden onset and abrupt cessation. The optic discs were normal, as was the VA. Neuroimaging revealed a pineal cyst. An EEG was normal and these episodes were ascribed to raised intracranial pressure due to presumed intermittent aqueductal occlusion. Neurosurgical cyst fenestration was performed without complications, and follow-up brain MRI showed no hydrocephalus. Later she would also be diagnosed with migraine with visual aura.

One year later, VA was 0.5 bilaterally, while near VA was 0.8 for both eyes. For the first time she received glasses (+ 2.75 sph.) for the previously uncorrected mild hyperopia. The optic discs were reported to be normal.

At age 7 years she started complaining of gradual worsening of vision on both eyes. Ophthalmological examination at age 8 years 2 months revealed VA of 0.5 in the right eye and 0.4 in the left eye. Funduscopy showed bilateral mild temporal optic disc pallor. Ocular coherence tomography (OCT) revealed significant bilateral temporal reduction of retinal nerve fiber layer (RNFL) thickness and some degree of superior and inferior swelling (pseudo-papilledema). This prompted genetic testing of the mitochondrial DNA at age 8 years 6 months, which disclosed a m.14484T>C homoplasmic mutation in the MT-*ND6* gene, previously also detected in her healthy mother and male maternal relatives affected with LHON.

**Fig. 1:**
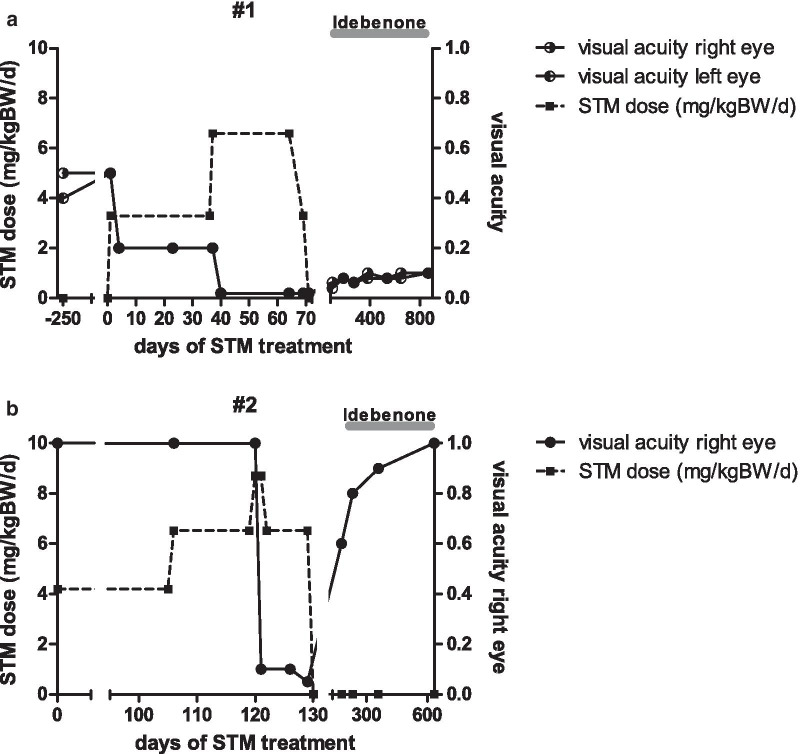
Timeline of STM dosage and visual acuity in two unrelated juvenile patients with LHON. **a** Patient #1 already had visual impairment with visual acuity of 0.5 before start of STM treatment. Shortly after initiation of antiepileptic medication with STM 3.3 mg/kg BW per day the patient complained of further visual impairment with visual acuity of 0.2 as assessed 3 weeks later. Due to persistently recurring seizures STM dosage was increased to 6.6 mg/kg BW per day. A few days later (9 weeks after start of STM) the girl complained marked further visual decline with visual acuity of 0.02. Hereupon STM was stopped. Under treatment with idebenone a slight recovery of visual acuity was observed in the following months. **b** Patient #2 had no history of previous visual impairment. Antiepileptic treatment with STM was started with 4.2 mg/kg BW per day. Due to persistently recurring seizures the dosage was increased in two steps to 8.7 mg/kg BW per day after 4 months. The next day the boy complained marked visual loss on his right eye, visual acuity was 0.1 in the subsequent ophthalmologic examination. Thus STM was stopped and a treatment with idebenone was initiated. In the further course a complete recovery of visual acuity could be observed

At age 8 years 10 months she presented with a 4-weeks history of recurrent focal seizures. These started with an aura of feeling unwell, reddening of the face, headache, predominantly on the right side, numbness of lips and tongue, slurred speech, and blurred vision. Her mother reported myoclonic jerks, tonic stiffening of one or both legs and dystonic posturing of her feet during the seizures, which lasted for up to 60 min. While she remained awake and responsive throughout the seizure, she had complete retrograde amnesia for the duration of each seizure. She complained of postictal nausea and vertigo. General examination was normal, and neurological examination was unremarkable, other than the bilateral visual loss, central scotoma and optic atrophy. Repeat neuroimaging did not show new findings. EEG revealed multifocal epileptic discharges. A diagnosis of cryptogenic focal epilepsy was made, and antiepileptic treatment was started, with STM with 3.3 mg/kg BW per day. Shortly thereafter she complained of worsening of the visual impairment. Three weeks after starting STM, the VA was 0.2 in both eyes. STM treatment led to a marked reduction of seizure frequency, but not to seizure freedom, and STM dosage was increased, 5 weeks later, to 6.6 mg/kg BW per day. After just a few days on this dose of STM, the girl complained of significant worsening of the visual decline on both eyes. The ophthalmological examination 9 weeks after starting STM showed “off-chart” VA with finger counting bilaterally (timeline shown in Fig. 1a). Funduscopy showed bilateral optic atrophy, with stable findings in RNFL-OCT.

Ten weeks after start STM was discontinued and replaced by levetiracetam (LEV). After stopping STM, the girl and her mother reported an improvement of vision in both eyes. Three weeks after stopping STM, VA was 0.063 in the right eye and 0.04 in the left eye, and 3 months later VA was 0.08 bilaterally (Fig. 1a). The patient had by then been started on idebenone 900 mg per day, as part of a named-patient program for patients with LHON, and she took idebenone for 4 years. She remained seizure free on LEV monotherapy, which was then stopped at age 12 years. The VA at last follow-up was 0.05 in both eyes.

#### Subject 2

This currently 15-year-old boy, unrelated with subject 1, is the first of two sons of healthy non-consanguineous parents. His younger brother is healthy. After uneventful pregnancy he was born at term with normal weight, length and head circumference. His motor, verbal, and cognitive development was normal. At age 11 years he presented with a 12 months history of recurrent focal seizures. He had two seizure types, one with behavioral disturbance and amnesia, the other with vertigo, headache, pallor, eye deviation, speech arrest, and loss of muscle tone. Seizures occurred mostly twice per week, with an unusual long duration of 15 to 120 min. Physical examination was normal, apart from overweight (body mass index 23.4 kg/m^2^; + 1.70 z, 96th centile). EEG showed right temporal epileptic discharges. MRI of the brain was normal. A diagnosis of a cryptogenic, possibly genetic focal epilepsy was made. Antiepileptic treatment with STM was started with 4.2 mg/kg BW per day. Four months later, at age 11 years 4 months, the dosage was increased to 8.7 mg/kg BW per day, due to persistent recurring seizures. Without any other unusual preceding event, the next day the boy complained marked visual loss on his right eye, without accompanying pain or other symptoms. He was admitted to his local pediatric hospital. Ophthalmological examination revealed VA of 0.1 and relative afferent pupillary defect (RAPD) on the right eye as well as mild bilateral optic nerve swelling (timeline shown in Fig. 1b). Neuroimaging was normal. A diagnosis of optic neuritis was made, and he was treated with a methylprednisolone pulse for 3 days, without improvement. STM was discontinued. At follow-up in the epilepsy outpatients clinic 10 days later antiepileptic treatment with LEV was started. Visual evoked potentials (VEP) using pattern shift stimulation were normal from the left eye and not elicitable from the right eye. Further laboratory tests failed to show evidence for an inflammatory or autoimmune optic neuritis. Genetic testing of the mitochondrial DNA revealed a homoplasmatic m.3460G>A mutation in the MT-*ND1* gene, yielding the diagnosis of LHON. This mutation was later also found in his mother and his younger brother. Treatment with idebenone, 900 mg per day, was started and continued for 2 years. VA on the right eye gradually recovered to 1.0 at follow-up investigations (Fig. 1b). At most recent follow-up, at age 15 years 8 months, VA and VEP were normal, as were color vision and visual field, but a mild right-sided structural RNFL deficit was seen in OCT. The boy has remained seizure free for the past 2 years.

### Real-time respirometry in fibroblasts from LHON patients and a healthy control

**Fig. 2:**
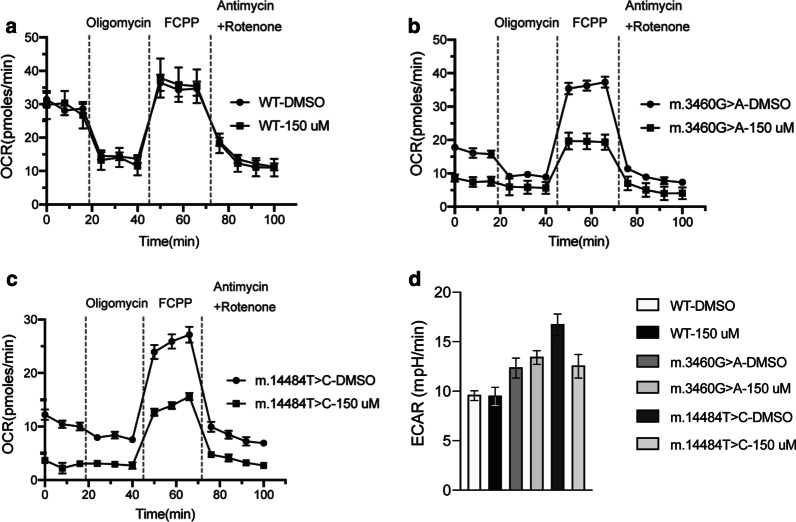
Real-time respirometry in fibroblasts of LHON patients. Respiratory chain activity was assessed by Real-Time respirometry (Seahorse measurement) in primary fibroblasts derived from **a** a healthy control, **b** a patient carrying the m.3460G>A mutation, and **c** a patient carrying the m.14484T>C mutation. Cells were incubated in the presence of DMSO or STM 150 µM for 3 h before the measurement (Mean, SEM, n = 6). OCR, oxygen consumption rate. **d** Extracellular acidification rates (ECAR) of primary fibroblasts (as in panels **a**–**c**) after uncoupling respiration with FCCP (Mean, SEM, n = 6)

Fibroblasts from a healthy control did not show significant differences in their respiration rates when treated with 150 µM STM or with vehicle control (dimethyl sulfoxide, DMSO) (Fig. 2a). In contrast, fibroblasts carrying the m.3460G>A or the m.14484T>C mutation showed a drastic reduction of respiration rate when treated with STM compared to vehicle control (DMSO), particularly when respiration was uncoupled with carbonyl cyanide-4-(trifluoromethoxy)phenylhydrazone (FCCP) (Fig. 2b, c).

Extracellular acidification rates (ECAR) were found to be generally higher in LHON fibroblasts compared to control cells, probably caused by enhanced glycolysis due to mitochondrial dysfunction (Fig. 2d). However, STM treatment did not significantly increase ECAR in any of the cell lines studied.

## Discussion

We describe two unrelated patients, an 8-year-old girl (subject #1) and an 11-year-old boy (subject #2), who suffered acute visual loss in close chronological connection with starting STM treatment for cryptogenic focal epilepsy. Subject #1 had already a mild bilateral visual impairment and an established LHON diagnosis (associated with the m.14484T>C mutation) before initiation of STM treatment. Her visual acuity markedly decreased within a few days after starting of STM treatment, and worsened further within a few days of increasing the STM dosage.

Subject #2 had acute monocular painless visual loss the day after increasing the STM dosage, at age 11 years 4 months. He was later diagnosed with LHON associated with the m.3460G>A mutation. In both subjects, there was no evidence for any other environmental factors known to trigger visual loss in LHON mutation carriers.

The question of whether these observations reflect a causal relationship between STM intake and triggering the onset of LHON-associated visual loss, or whether this is mere temporal coincidence, is difficult to assess.

LHON (MIM #535000), the most common mitochondrial disorder, is a maternally inherited disease characterized by acute or subacute painless visual loss [14–17]. Visual failure occurs most often in young adult life, with a clear predominance of affected males (5:1 male to female ratio) [17]. In the acute phase, patients describe loss of colour vision and visual blurring in the central visual field in one eye. Visual field testing shows a progressively enlarging centrocecal scotoma, and in most cases, there is severe visual loss, with markedly reduced VA. Usually within 2 or 3 months, the second eye is similarly affected, but bilateral onset of visual loss occurs in about 25% of cases. Later optic atrophy develops, with lifelong bilateral visual loss, leaving most patients legally blind [15].

The vast majority of European and North American patients carry one of three pathogenic mutations of the mitochondrial DNA (m.11778G>A, m.3460G>A, m.14484T>C), which lead to dysfunctional complex I of the mitochondrial respiratory chain [15]. This results in a defect of adenosine triphosphate (ATP) synthesis and an enhanced production of reactive oxygen species (ROS), leading to dysfunction and loss of a proportion of retinal ganglion cells [16].

Genotype–phenotype correlations have been recognized, and the m.14484T>C variant in the MT-*ND6* gene is related to a most favorable long-term visual outcome [15].

Penetrance of LHON is incomplete, visual loss arises in only ca. 50% of males and ca. 10% of females carrying one of the three primary LHON mutations. Occasional discordance of LHON in monozygotic twins indicates that environmental factors may have an impact on penetrance [18]. Factors triggering visual loss in LHON mutation carriers include smoking, heavy intake of alcohol, and raised intraocular pressure [19–21]. Poor nutrition, psychological stress, physical trauma, and acute illness have been blaimed as well [18, 19].

The lifetime risk for visual failure in individuals carrying the homoplasmic mutation m.14484T>C of the mitochondrial DNA was estimated to be 47% in males and 8% in females [22]. The mean age at onset of visual loss in females was 17.7 years, with a range of 11 to 21 years, in one case series [22]. The onset of visual impairment already at age 7 years observed in subject #1—more than 1 year before STM treatment was started—is earlier than what would be expected in the literature. But for this patient a critical worsening of the visual loss occurred in close temporal relation with starting treatment with STM and increasing the dose of STM.

The lifetime risk for visual failure in individuals with the homoplasmic mitochondrial DNA pathogenic variant m.3460G>A was in one study reported to be 32% in males and 15% in females with a median age of onset of 20 years in males [15]. A second study described a risk of 49% in males and 28% in females, with a median age of onset of 22 years (standard deviation 13.6 years) in males [17]. The range of age of onset was not provided in these studies. Subject #2 suffered visual loss at age 11 years 4 months, relatively early in life. As for subject #1, visual loss occurred in close temporal relation with a dose increase of STM treatment.

Apart from visual impairment, most LHON patients are otherwise healthy. However, occasional associations with neurological, cardiac, and skeletal features were reported in some patients with LHON. Neurologic features described to occur in LHON more commonly than in the general population include postural tremor, peripheral neuropathy, nonspecific myopathy, and movement disorders. A multiple sclerosis-like condition (or “Harding´s disease”) was observed in some subjects with LHON, mostly women [15, 23]. A recent nationwide study from Denmark revealed an increased incidence for stroke, demyelinating disorders, dementia, and epilepsy in individuals with LHON, but not in their family members [24]. Focal epilepsy in childhood, as observed in the two subjects reported here, is unusual to occur in patients with LHON. We can only speculate whether LHON and epilepsy in these two subjects are caused by a unique pathomechanism or whether they represent two independent conditions.

A deleterious effect on mitochondrial function has been shown, or is pathophysiologically possible, for a wide range of medications [25, 26]. Several substances known to interfere with the function of the mitochondrial respiratory chain are under suspicion of triggering LHON in mutation carriers. These include antibiotics such as ethambutol, chloramphenicol, linezolid, aminoglycosides; antiretroviral drugs; as well as cyanides, methanol, pesticides, and phosphodiesterase type 5 inhibitors [25–30].

Another mitochondrial carbonic anhydrase inhibitor used for antiepileptic treatment, topiramate (TPM), was reported to have triggered visual loss in an adult patient carrying the LHON m.11778G>A mutation, who had received TPM for temporal lobe epilepsy [31].

The pathomechanisms of triggering of onset of visual loss onset in LHON mutation carriers by carbonic anhydrase inhibitors is difficult to elucidate. Previously reported animal experiments point to an impairment of oxidative phosphorylation caused by metabolic acidosis [32–34]. Thus, the metabolic acidosis known to occur under medication with carbonic anhydrase inhibitors is a possible explanation leading to impairment of mitochondrial function, resulting in visual loss in LHON mutation carriers. However, while an impact of STM-associated metabolic acidosis on mitochondrial function seems theoretically possible, reports of significant acidosis under STM treatment are rare [8, 12], and it was shown that urinary pH remains in the normal range under STM treatment [35]. Furthermore, in this study we did not observe a significant increase of extracellular acidification rates in LHON fibroblasts compared to control cells when treated with STM. Thus, STM-associated metabolic acidosis may possibly play only a minor role in triggering onset of visual failure in LHON.

Mitochondria are impermeable to HCO3^−^. The two mitochondrial carbonic anhydrase isoforms VA (CA-VA) and VB (CA-VB) are responsible for providing HCO3^−^ to essential mitochondrial enzymes [Carbamoyl-phosphate synthase (CPS1), pyruvate carboxylase (PC), propionyl-CoA carboxylase (PCC), 3-methylcrotonyl-CoA carboxylase (3MCC)]. These enzymes catalyze the production of metabolic intermediates of the urea and Krebs cycle [36]. Thus, it is tempting to speculate that STM diminishes the production of reducing equivalents through the Krebs cycle, decreases ATP production and impairs oxidative phosphorylation. However, at this point we can not exclude that STM could also affect functionality of the OXPHOS system in a more direct manner. Further analyses will be required to address this question.

In fact, we provide evidence from real-time respirometry in LHON fibroblasts that STM drastically deteriorates their respiration rate when treated with STM compared to vehicle control. We conclude that STM has a deleterious effect on cells with an already compromised mitochondrial function, which may explain the onset or worsening of visual loss under STM treatment.

## Conclusions

Taken together, we describe a relationship between STM medication and onset of visual failure in these two subjects with LHON. We conclude that antiepileptic medication with STM, a carbonic anhydrase inhibitor, may pose a risk for visual loss in LHON mutation carriers and should be avoided in subjects known to carry a pathogenic mutation of mitochondrial DNA associated with LHON. Before starting STM medication in patients with seizures we recommend asking for a family history of LHON or unexplained blindness.

Awareness of this possible connection between STM and triggering or worsening visual loss in LHON mutation carriers, especially among pediatricians and pediatric neurologists, may improve the outcome for some mutation carriers of a LHON-associated mutation, and further shape the profile of adverse effects of this widely used antiepileptic drug.

## Methods

### Subjects

Clinical and laboratory data of two unrelated patients seen in our outpatient pediatric epilepsy clinic were extracted from the patients´ files.

### Biochemical investigations in fibroblasts

For ethical reasons we obviated skin biopsies in the two children reported here and, instead, used fibroblasts from two adult LHON patients carrying the same mutations as in our patients, the m.14484T>C missense mutation in the MT-*ND6* gene and the m.3460G>A missense mutation in the MT-*ND1* gene, respectively. These fibroblasts had been established from patients´ skin biopsies, cultured and maintained in the Friedrich-Baur Institute, Munich, Germany, for research purposes. Additionally, we retrieved fibroblasts from a healthy control which were stored for research purposes as well. We obtained written informed consent from these adult subjects. All studies were performed in accordance with the Declaration of Helsinki protocols. The studies were approved by the local institutional ethics boards.

### Cell culture

Primary fibroblasts were cultured in Dulbecco's Modified Eagle Medium (DMEM), supplemented with 10% (v/v) Fetal Bovine Serum (FBS), 1 mM sodium pyruvate, 2 mM L-glutamine and 50 μg/mL uridine under a 5% CO_2_ humidified atmosphere at 37 °C.

### Real-time respirometry

OCR and ECAR were measured with a XF96 Extracellular Flux Analyzer (Seahorse Bioscience, Billerica, MA, USA) as previously described [37]. After gelatin (1%) coating fibroblasts were seeded at 22.000 cells/well. Cells were incubated with 150 µM STM or DMSO for 3 h. Basal respiration was measured in DMEM supplemented with 1 mM pyruvate and 10 mM glucose after calibration at 37 °C in an incubator without CO_2_. Periodic measurements were performed and OCR was calculated from the slope of change in oxygen concentration over time. Metabolic states were measured after subsequent addition of 3 μM oligomycin, 1 μM FCCP, 1 μM antimycin A, and 2 μM rotenone. ECAR were measured after addition of FCCP, and the results of 3 measurements were averaged.

## Data Availability

The datasets used and/or analysed during the current study are available from the corresponding author on reasonable request.

## Abbreviations

3MCC: 3-Methylcrotonyl-CoA carboxylase
ATP: Adenosine triphosphate
BECTS: Benign epilepsy of childhood with centrotemporal spikes
BW: Body weight
CA: Carbonic anhydrase
CPS1: Carbamoyl-phosphate synthase
DMEM: Dulbecco's Modified Eagle Medium
DMSO: Dimethyl sulfoxide
ECAR: Extracellular acidification rate
FCCP: Carbonyl cyanide-4-(trifluoromethoxy)phenylhydrazone
FBS: Fetal Bovine Serum
LEV: Levetiracetam
LHON: Leber hereditary optic neuropathy
OCR: Oxygen consumption rate
OCT: Ocular coherence tomography
PC: Pyruvate carboxylase
PCC: Propionyl-CoA carboxylase
RAPD: Relative afferent pupillary defect
RNFL: Retinal nerve fiber layer
ROS: Reactive oxygen species
STM: Sulthiame
TPM: Topiramate
VA: Visual acuity
VEP: Visual evoked potentials
